# Improving Performance of Bluetooth Low Energy-Based Localization System Using Proximity Sensors and Far-Infrared Thermal Sensor Arrays

**DOI:** 10.3390/s25041151

**Published:** 2025-02-13

**Authors:** Vitomir Djaja-Josko, Marcin Kolakowski, Jacek Cichocki, Jerzy Kolakowski

**Affiliations:** Institute of Radioelectronics and Multimedia Technology, Warsaw University of Technology, 00-661 Warsaw, Poland; marcin.kolakowski@pw.edu.pl (M.K.); jacek.cichocki@pw.edu.pl (J.C.); jerzy.kolakowski@pw.edu.pl (J.K.)

**Keywords:** indoor localization, hybrid positioning system, BLE, proximity sensor, thermal sensor array, distance measurements, angle estimation, particle filter

## Abstract

This paper presents the concept of a hybrid positioning scheme using results from a Bluetooth Low Energy (BLE)-based system and additional infrared (IR) devices: proximity sensors and far-infrared thermal sensor arrays. In the proposed solution, the IR sensors operate independently from the BLE subsystem. Their output (the distance to the localized person and the angle between the sensor axis and the person’s location) is periodically used to improve the positioning accuracy. The results from both parts of the system are fused using a particle-filter-based algorithm. The proposed concept was tested experimentally. The initial tests established that both the proximity (VL53L5CX) and array (MLX90640) sensors allowed for angle estimations with a mean accuracy of about a few degrees. Using them in the proposed hybrid localization scheme resulted in a mean positioning error decrease of several centimeters.

## 1. Introduction

### 1.1. Background

According to the European Commission’s Long-Term Care Report [1], the population aged 65+ will increase to 108.5 million in 2030 and will reach a 24.1% share of the total population. It is estimated that 19.2% of this group will require long-term care, which will involve different services designed to meet their health and personal care needs when they can no longer perform everyday activities. Evaluations of older adults’ performance in their activities, their activity patterns, and the time they expend can be used to evaluate a person’s health and trigger interventions in cases of behavioral changes or major events [2].

Although activity recognition systems can use different technologies, solutions based on inertial sensors are the most popular [3]. Recent advancements in smartphones, smartwatches, and other wearables equipped with multiple sensors have made these devices especially useful [4]. Most activity detection algorithms rely on machine learning, primarily neural networks [5,6].

Activity recognition systems can be enhanced through the use of indoor positioning systems. Although location data on their own can give some insights into the current activity [7], the system output is usually used as an additional context enhancement in potential analyses.

There are two leading types of indoor radio positioning systems: ultra-wideband (UWB) and narrowband (usually based on Bluetooth or Wi-Fi). Bluetooth Low Energy (BLE) devices are especially popular in the AAL (Active Assisted Living) domain. Practically all modern wearables are equipped with BLE modules enabling communication with external devices, e.g., smartphones and tablets. Due to the popularity of BLE, the cost of systems that utilize it is low compared with that of other technologies.

Unfortunately, the typical BLE-based positioning systems are not very accurate. Positioning algorithms that use Received Signal Strength (RSS) values suffer from significant variations in signal levels in multipath environments. Positioning errors of several meters are common in these implementations. In such cases, even the identification of the room that a person is visiting may not be reliable. Recent advances in the standards for BLE, especially those introducing angle of arrival (AOA) measurements, may improve the localization accuracy but at the cost of more complex (and expensive) positioning system infrastructure. Besides AOA measurements, the accuracy of BLE-based positioning systems can be enhanced by implementing hybrid algorithms that combine typical RSS or angle of arrival measurements with additional data acquired using other sensors and systems.

Many AAL devices are equipped with inertial sensors. However, using them to support an indoor positioning system may be problematic because of the need for complex calculations, requiring a high energy consumption, which can be hard to implement in wearable devices. Processing the results in the central unit requires transferring the raw (or preprocessed) results to the system controller, further draining the device battery.

The research presented in this paper is a continuation of the work undertaken in [8], where the potential of a hybrid BLE–proximity sensor system was investigated in positioning tests. This paper extends this concept and provides the following novel contributions:

- A novel hybrid positioning scheme utilizing a BLE-based radio system, infrared proximity sensors, and far-infrared thermal sensor arrays is proposed;
- A novel particle-filter-based algorithm fusing the positioning data obtained using the BLE and infrared sensors is proposed;
- A far-infrared array sensor (MLX90640, Melexis, Mechelen, Belgium) and an infrared proximity sensor (VL53L5CX, STMicroelectronics, Geneva, Switzerland) are tested separately in a person positioning scenario;
- The proposed concept and algorithm are tested under laboratory conditions using a BLE-based positioning system.

The implementation of the proposed hybrid positioning scheme improved the positioning accuracy of the BLE-based positioning system. The proposed approach can be used to upgrade positioning systems regardless of the technology used for positioning. It operates in an unobtrusive manner and does not interfere with devices already owned by the user.

### 1.2. The State of the Art in IR-Based Systems

Most works on indoor localization through IR sensing have been related to passive infrared (PIR) sensors, and only some have utilized thermal images. PIR sensors are simpler and provide fewer data than thermal images, but they still allow for coarse localization after proper processing.

In [9], D. Hauschildt and N. Kirchhof proposed a solution that utilized multiple PIR sensors consisting of an array of thermopiles placed in the corners of a room. The solution was tested, and localization errors in the 0.09 to 0.68 m range were achieved. The same authors introduced a modified approach in [10] combining PIR localization with a time difference of arrival (TDOA) ultrasound system, which proved to have a better overall accuracy. R. C. Luo, O. Chen, and others proposed a solution in [11,12,13] that fused PIR sensors with ZigBee radio modules in a so-called WPIR (Wireless and Pyroelectric Sensor) system. The solution was tested, and a mean error of 0.73 m was achieved. In [14], S. Narayana and others presented a comprehensive overview of the state of the art regarding using thermopiles and PIR sensors for indoor localization. They cited the works of B. Mukhopadhyay and others [15], who used four PIRs in a room 7 by 7 m in size. Other cited works [16,17,18,19,20] included the use of multiple thermopiles (from one up to five) mainly for people localization and tracking, but also for fall detection [16] or simple occupancy detection [17]. All of the cited works carried out testing in single rooms and achieved a sub-meter accuracy. This proves that the limitation of IR-based systems, which is the need for line-of-sight (LOS) conditions between the localized person and the sensor, may indeed be a problem when deployments in more extensive areas are needed. G. Petrova and others presented a detailed review of the utilization of IR array sensors for people localization and activity detection in [21]. B. Zhou and others, in [22], presented DarkLoc, the idea of attention-based indoor localization using thermal images and convolutional neural networks (CNNs). These authors achieved results with errors ranging from roughly 0.5 m to 1 m depending on the number of images used to train the model. An improved version of this system, called DarkLoc+, was presented in [23].

Some authors have also worked with infrared sensors capable of estimating the distance and angle. T. Salzmann and M. Magno presented a method using thermal infrared sensors (TMOSs) in [24]. T. Aytac and B. Barshan proposed another approach in [25] that included an active sensor, which combined an infrared emitter and receiver and allowed for estimations of the distance and angle with an accuracy of roughly 1 cm and 2°, respectively.

### 1.3. The State of the Art in Hybrid BLE-Based Systems

Different methods are used to improve the performance and robustness of BLE-based localization systems. In the following analysis of the state of the art, emphasis was placed on solutions that introduced additional sensors (besides the inertial measurement unit (IMU) sensors incorporated into the localized tags) into the BLE localization system.

N. Kuxdorf-Alkirata and D. Bruckmann proposed a combined BLE- and IR-based system in [26], where RSS measurements of the BLE signals were utilized for position estimation and a Grid-EYE 8 × 8 array thermal sensor was used for dynamic obstacle detection. Proximity sensors can also be used to improve the performance of RSS localization systems. In [27], data fusion of the results from laser infrared sensors and a BLE-based positioning system allowed for improvements in the localization accuracy. Information on the distance to the object was fused using a Kalman filter. The work presented in [26,27] is promising, as it proves that the usage of IR-based and laser infrared sensors in conjunction with a BLE-based localization system is feasible.

In [28], a fused indoor localization method that makes use of binary sensors, a capacitive floor, and the signal strength received at a smartwatch coming from BLE beacons is proposed. A particle filter was used in the location engine. In [29], the authors presented another hybrid approach where BLE and ultrasound technologies were utilized. The proposed method is based on the augmentation of the BLE beacons with synchronized ultrasound transmitters. Performed system tests proved that the localization accuracy achievable is within a few dozen centimeters.

Another approach involving BLE, acoustic, and Light Fidelity (Li-Fi) technologies is presented in [30]. The BLE subsystem is used for RSS-based ranging, the acoustic subsystem for angle estimation, and the Li-Fi subsystem for proximity detection. Performed tests showed an accuracy in the range of a few dozen centimeters.

The authors of [31] proposed a combined BLE and mmWave radar system for accurate localization. It utilized the AOA estimation introduced in BLE v5.1 and the mmWave FMCW (Frequency-Modulated Continuous Wave) measurements for heatmap creation and data fusion using a neural network. The achieved localization error was in the sub-meter range.

Another hybrid approach that utilizes a BLE subsystem and a camera worn by the localized person is presented in [32]. This solution uses a neural network to fuse the BLE RSS measurements with the data on the person’s presence obtained using the worn camera. Tests in the laboratory environment showed that the localization error for the proposed method was roughly 4 m for 90% of the results.

The structure of the rest of this paper is as follows. Section 2 and Section 3 are dedicated to a description of the proposed architecture and localization algorithms. The results of experiments comprising IR sensor measurements and positioning tests are presented in Section 4. This paper concludes with Section 5.

## 2. The BLE-IR Localization System

### 2.1. System Architecture

**Figure 1:**
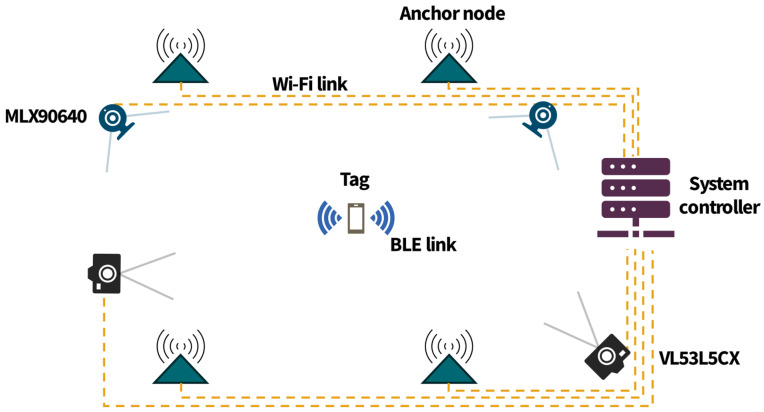
The architecture of the proposed BLE-IR localization system.

The proposed BLE-IR localization system consists of four major elements: localized tags that transmit BLE frames, a fixed infrastructure comprising anchor nodes acting as receivers, independently working IR sensors, and a system controller responsible for the position calculation. The proposed architecture is shown in Figure 1.

The tags send six BLE frames per second. They are received by the anchor nodes, which measure their RSS. Every second, the measurement results accumulated from each node are sent to the system controller. One of the anchor nodes serves as the reference node, which dictates the sequential number (SQN) of the transmitted frames to keep all of the anchor nodes synchronized.

The tags and anchor nodes share the same BLE module, BL652, from Laird Connectivity (now known as Ezurio) [33]. A more detailed description of the BLE subsystem in the localization system’s architecture is presented in [34].

In the proposed solution, the IR sensor can be an MLX90640 far-infrared thermal sensor array with a resolution of 32 × 24 pixels and a 110° field of view (FOV) [35] or a VL53L5CX multizone ToF sensor capable of measuring the distance in up to 64 zones (an 8 × 8 grid) at a range no greater than 4 m [36]. In either case, the sensors work independently of the BLE subsystem but are synchronized with it, so all of the measurement results are tied to the sequential numbers of Wi-Fi frames transmitted by the anchor nodes. The MLX90640 sensor captures thermal images which are processed to detect whether a person is present. If so, the angle at which the person is located relative to the vertical plane perpendicular to the sensor plane is estimated.

The VL53L5CX measurements are used to detect a person’s presence in front of the sensor and the distance to this person and to estimate the angle at which the person is located relative to the vertical plane perpendicular to the sensor plane. The data gathered by both sensors are fused with the RSS data, and the position is calculated.

### 2.2. Infrared Sensors

#### 2.2.1. The MLX90640 Angle Estimation Algorithm

**Figure 2:**
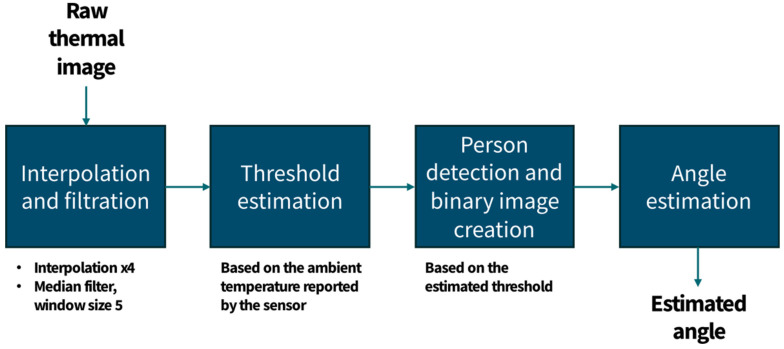
MLX90640 angle estimation algorithm.

**Figure 3:**
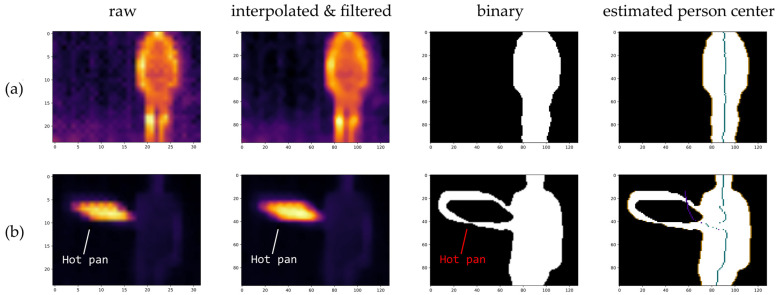
Illustration of the process of estimating the location of a person’s silhouette in (**a**) typical conditions and (**b**) near a large heat source. Estimation of the center of the person’s silhouette is performed in two phases; the results are marked in purple (first phase) and green (second phase).

The MLX90640 outputs low-resolution (32 × 24 pix) thermal images, which allows for the estimation of the angle between the sensor and the localized person. The algorithm for angle estimation is based on [37]. The algorithm flow is presented in Figure 2. An illustration of the data processing in the two scenarios is presented in Figure 3.

The first step consists of increasing the resolution of the thermal image to 128 × 96 by performing interpolation and median filtration. Initial tests proved that the best angle estimation results were achieved for four-time interpolation and filtering with a median filter with a window size equal to five.

In the next step, the lower and upper temperature thresholds are estimated for detecting a person’s presence. The sensor, besides the thermal image, returns the ambient temperature parameter (which may be slightly higher than the actual ambient temperature due to the heating of the sensor itself and the microcontroller used to control it). Although this value may be used as a benchmark for setting the lower threshold, another approach was adopted due to heating issues. In the proposed implementation, the lower threshold is obtained by computing the mean temperature of the image and adding 0.9 °C. If any of the pixels’ values in the thermal image are above this threshold, it is assumed that the person is present in the sensor’s FOV. The upper temperature threshold is necessary to eliminate false positives regarding warm objects in the camera’s view, such as heaters, computers, or cooking pots. In the implementation, an arbitrary value of 50 °C was chosen.

After estimating the lower and higher bounds, the image is thresholded to obtain a binary mask of the person’s silhouette. As presented in Figure 3b, the binary image may not always include only the person’s silhouette due to the presence of hot objects in the vicinity. Despite them being eliminated through thresholding, the heat residue may lead to the formation of loops in the image.

The final step consists of estimating the angle between the sensor and the detected person. Due to the possible issues caused by hot objects within the sensor’s field of view, this is carried out in two phases. In the first phase, the center of the person’s silhouette is estimated by analyzing the image simultaneously from its left and right edges, finding the silhouette’s outer contours, and then calculating the middle point in each pixel row. In the second phase, the image is analyzed from the inside of the silhouette towards the outside, and yet again, the midpoints are calculated for each row. The outcome is presented in Figure 3b. The results of the first phase are marked in purple, whereas the final result is marked in green. The silhouette’s edges are marked in orange.

The center of the person’s silhouette is calculated as the mean value of all of the row midpoints. Assuming that the image is interpolated by a factor of 4, that the raw horizontal resolution is equal to 32, and that the sensor’s FOV is equal to 110°, the angle (α) at which the person is seen by the sensor is calculated as in Equation (1):

(1) $$\alpha =-55^{o}+\frac{n}{32\cdot 4}\cdot 110^{o},$$

where 55° is half of the sensor’s FOV, and n is the index of the pixel estimated as the center of the person’s silhouette. It can be also noted that under these conditions, the angle estimation resolution (Δα) is as in Equation (2):

(2) $$∆\alpha =\frac{1}{32\cdot 4}\cdot 110{}^{\circ}=\frac{55}{64}{}^{\circ}≅0.86{}^{\circ}.$$

#### 2.2.2. The VL53L5CX Angle and Distance Estimation Algorithm

The VL53L5CX sensor performs distance measurements in up to 64 independent zones (an 8 × 8 grid) at a range of up to 4 m with a FOV of 45°. This allows for angle estimations with a resolution of roughly 5.6°. The angle determination algorithm is divided into three phases. It utilizes data from two additional matrices provided by the sensor—motion and status matrices—which are serialized for ease of operation.

**Figure 4:**
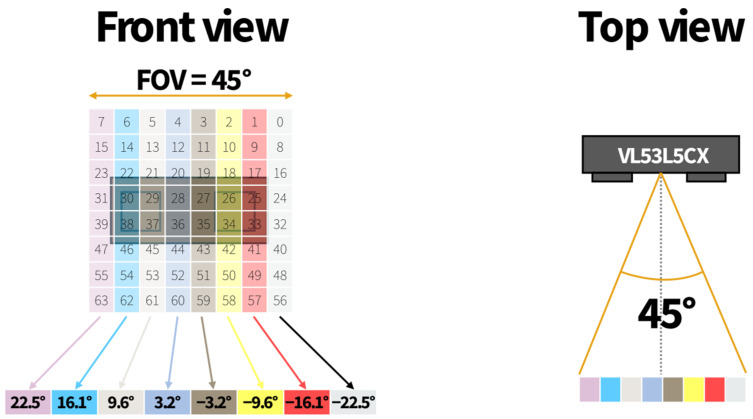
The VL53L5CX angle values for individual columns.

In the first step, the motion data are analyzed (the values in the cells are compared with the chosen threshold) to determine whether a moving person is within the sensor’s FOV. If so, indexes of the cells where motion is detected are saved. In the second step, the status values for those indexes are verified to check which of these indexes represents correct distance measurements. In the third step, all cells that contain proper distance measurements are grouped into columns. Each column represents a different angle, as shown in Figure 4. If the cells fall within separate columns, the angle is calculated as the mean value of the angles for individual columns.

## 3. Positioning Algorithms

The results from the sensors described in this paper can be used both in loosely and tightly coupled positioning schemes. In the loosely coupled scheme, the user’s location obtained based on the sensor’s distance and angle measurements is combined with the location obtained using the BLE-based system. In the tightly coupled approach, the sensor readings are processed alongside the signal power measurements in the internal algorithm steps.

The algorithms proposed in this paper are tightly coupled. As the basic algorithm implemented in the BLE-based system uses particle filtering, the sensor’s results were used to calculate the modified particle weights.

### 3.1. The Basic (BLE-Only) Localization Algorithm

**Figure 5:**
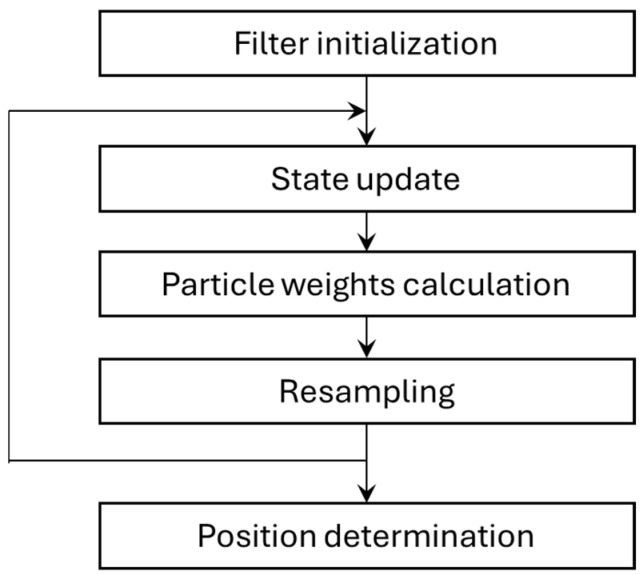
Particle filter processing steps.

The positioning algorithm used in the system is a particle filter (PF) [38] implementation. The algorithm consists of the typical steps presented in Figure 5:

Filter initialization consists of the random initialization of N particles (in our implementation, N equaled 2000), which are drawn from a four-dimensional uniform distribution. Each of them is described using a state vector

(3) $$x_{k}^{\left(i\right)}=\left[x\;v_{x}\;y\;v_{y}\right],$$

where x, y are the particle coordinates distributed over the monitored area, and $v_{x}$, $v_{y}$ are the velocity vector components in the −0.1 to 0.1 m/s range.

In the state update step, the current states (at the moment k) of the particles are estimated using the following:

(4) $$x_{k}^{\left(i\right)}={Fx}_{k-1}^{\left(i\right)}+n_{k-1},$$

where F is the matrix describing the uniform motion of the localized object, and the $n_{k-1}$ vector is a normally distributed noise component vector introducing additional differences between the particles.

The particles are assigned weights, which describe the degree to which they fit the measurement results. The weight of the i-th particle is calculated as follows:

(5) $$w_{i}=\eta {\cdot w}_{m,i}{\cdot w}_{g,i},$$

(6) $$w_{m,i}=\frac{1}{\left(\sqrt{\frac{\sum_{n}{\left(z_{k,n}-h_{k,n}\left(x_{k}^{\left(i\right)}\right)\right)}^{2}}{len\left(z_{k}\right)}}\right)},$$

(7) $$h_{k,n}\left(x_{k}^{\left(i\right)}\right)=\left[P_{1}\left(d_{1}\right)\ldots P_{n}\left(d_{n}\right)\right].$$

The $w_{g,i}$ component specifies whether the particle is in the valid area (if not, its value is 0; else, it is 1); η is the scaling factor assuring that the weights of all of the particles sum to one. The $w_{m,i}$ component describes how close the measurements performed in the system stored in the vector $z_{k}$ (RSS results) are to those estimated for the particle location $h_{k,n}\left(x_{k}^{\left(i\right)}\right).$ In the BLE-only positioning, the estimated measurement values are calculated using the exponential path loss model as in Equation (8):

(8) $$P\left(d\right)=P\left(d_{0}\right)-10\cdot n\cdot log\left(\frac{d}{d_{0}}\right),$$

where d is the distance between the particle and the anchor, n is the propagation constant, and power ${P(d}_{0})$ is the signal power measured at distance $d_{0}$. In our implementation, $d_{0}$ was 1 m, while n and ${P(d}_{0})$ were obtained experimentally and equaled 3.4 and −44, respectively.

The last step of the PF iteration is resampling, which consists of drawing a new set of particles with their probability depending on the weight values. The weighted average of the particles is an estimate of the object’s location.

### 3.2. The Proximity-Sensor-Based Localization Algorithm

The proximity sensor provides information on the distance to the detected object and the relative bearing (angle). The sensor’s range is relatively short, so the localization accuracy with the sensor is typically better than that in BLE-based systems. Therefore, when in the sensor range, the RSS measurements are replaced with measurements from the proximity sensor. The weights are still computed using (5–6), but now

(9) $$z_{k}=\left[x_{s,1}y_{s,1}\ldots x_{s,n}y_{s,n}\right],$$

(10) $$h_{k,n}\left(x_{k}^{\left(i\right)}\right)=\left[x_{k}^{\left(i\right)}\left[0\right]x_{k}^{\left(i\right)}[2]\ldots x_{k}^{\left(i\right)}\left[0\right]x_{k}^{\left(i\right)}[2]\right],$$

where $x_{s,i},y_{s,i}$ are the user coordinates derived based on measurements from the i-th IR proximity sensor. The measurement model $h_{k,n}\left(x_{k}^{\left(i\right)}\right)$ is a simple repetitive extraction of the particle’s x, y coordinates to match the length of $z_{k}$.

### 3.3. The Hybrid BLE–Far-Infrared Thermal Sensor Array Localization Algorithm

**Figure 6:**
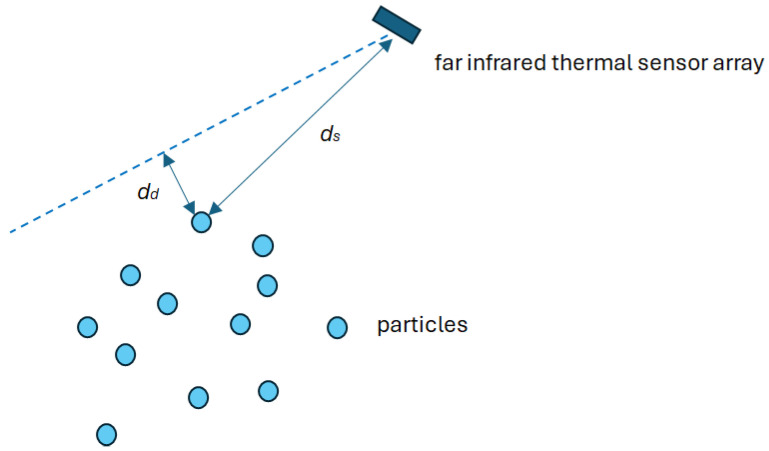
Particle weight components.

The far-infrared thermal sensor array’s readings can be used to evaluate the angle between the sensor and the localized person. When in the range of the sensor, the particle weights are updated based on Equations (11)–(13):

(11) $$w_{i}=\eta \left({\alpha \cdot w}_{m,i}+{\beta \cdot w}_{dd}{+\gamma \cdot w}_{ds}\right){\cdot w}_{g,i},$$

(12) $$w_{dd}=\sum_{1}^{n}\frac{1}{d_{d}},$$

(13) $$w_{ds}=d_{s}^{2},$$

where $w_{m,i}$ is a component describing how well the particles fit the BLE power measurement results (6); $w_{dd}$ describes how close the angle between the particle and the sensor is to that reported when calculating the reciprocal of the distance $d_{d}$ to the line using the sensor’s location and the detected object (Figure 6); and $w_{ds}$ accounts for the lower detection accuracy for the person’s silhouette at longer distances; α,β,γ are scaling factors (in the proposed implementation, their values are 100, 0.04, and 0.1, respectively).

## 4. Experiments

### 4.1. The Test Setup

The proposed concept was verified using experiments. Two types of tests were performed:

- Static tests, solely for thermal and ToF sensors to evaluate the proposed angle estimation methods and to calibrate the orientation of the IR sensors;
- Dynamic, full-system tests, where the localized person follows a predefined path, to make the test conditions as close as possible to real-life use cases.

**Figure 7:**
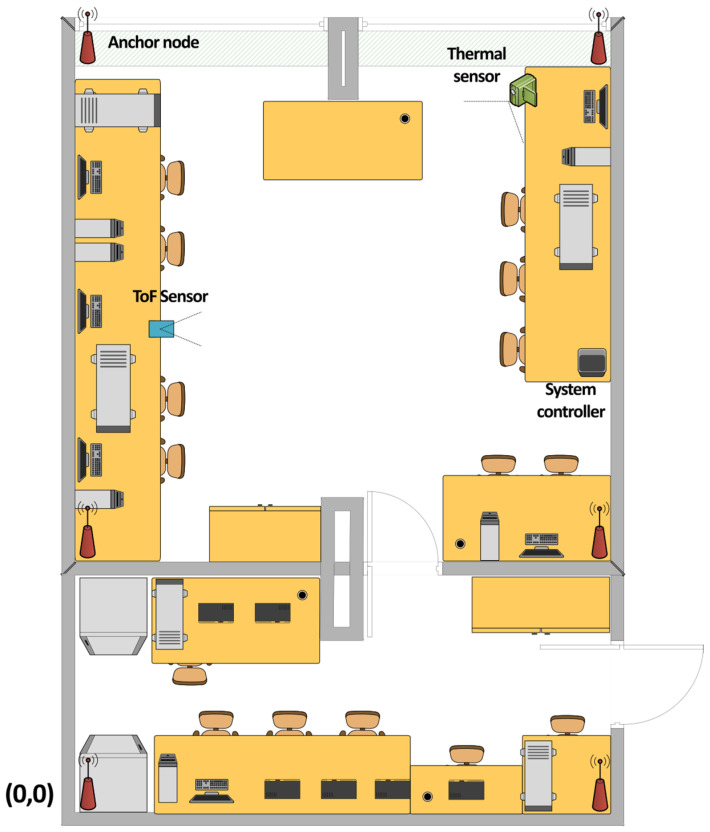
Test setup for measurements.

All tests were performed in a laboratory room, 6 by 6 m in size, with the windows facing north, fully furnished, and filled with different electronic equipment (Figure 7). For the full system tests, three different configurations were tested—the BLE localization system and its hybrid versions, using the MLX90640 and VL53L5CX sensors.

The BLE anchor nodes were placed in the corners of the room near the ceiling to provide unobstructed propagation conditions, with the two anchor nodes placed in the adjacent room. The thermal sensor was placed in the corner of the room to achieve the best possible coverage. Due to its limited range, the ToF sensor was placed on a table, facing the center of the room. The origin of the coordinate system was such that the point (0,0) was in the lower-left corner of the smaller room.

During the tests, a constant temperature of 23 °C and 40% humidity were maintained in the room. Both tests were carried out on cloudy days.

### 4.2. Evaluation of MLX90640

**Figure 8:**
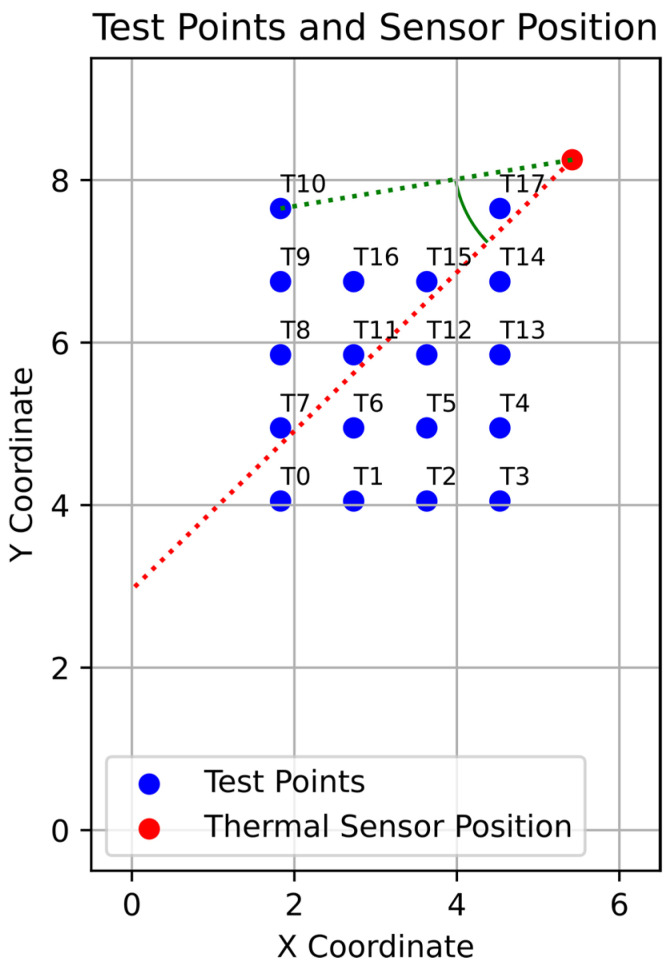
Test setup for static measurements of the MLX90640 far-infrared thermal sensor array.

The initial MLX90640 tests were performed statically at 18 different test points, as shown in Figure 8. One exemplary angle for test point T10 is marked for clarity. The red line shows the middle of the sensor’s FOV.

A person stood for around ten seconds at each test point, translating to roughly 40 angle measurements. To align the thermal sensor array’s measurement plane with its surroundings, measurement T1 was treated as a calibration, which meant that the offset between the mean angle and the actual angle was calculated and later subtracted from all of the following measurements.

In Table 1, the results obtained are presented.

**Figure 9:**
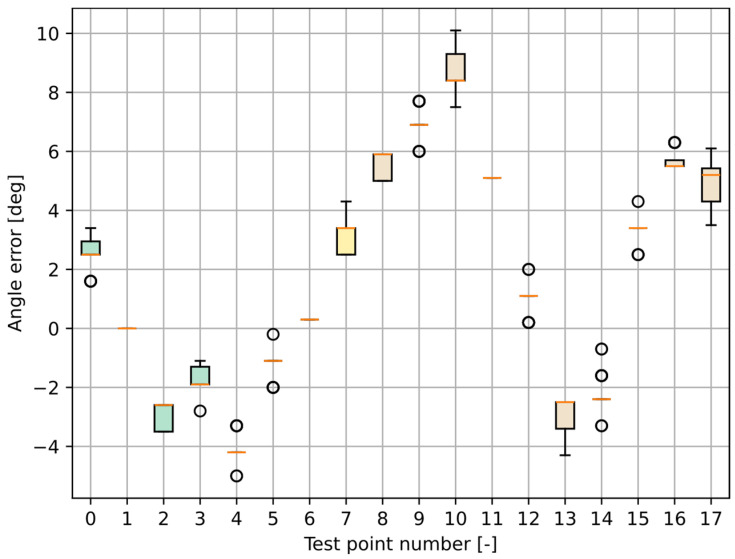
Boxplots of the MLX90640 static measurement results.

A boxplot of the angle estimation errors is shown in Figure 9.

As can be seen, the results are relatively good, with the absolute mean angle estimation error being smaller than 10° for all test points and smaller than 5° for most of them and the standard deviation being smaller than 1°. Distance does not affect the angle estimation; however, a pattern may be observed regarding the influence of the real angle. Better results are achieved for the test points placed directly in front of the sensor, and the more the point deviates to the side, the greater the error. It is hard to predict clearly how these errors may affect the positioning accuracy, as it also depends on other factors. Generally, four primary sources of errors may be identified:

- The incorrect alignment of the sensor—It is hard to perfectly adjust it to point precisely in the desired direction;
- Incorrect detection of a person’s presence—The person may be wrongly detected, or there may be other warm objects in the image that may be perceived as a person by the algorithm, which leads to incorrect angle estimation;
- The angular width of a person is not infinitely small—A person always has an angular width when standing in front of the sensor, which becomes bigger the closer the person is. It is hard to estimate the perfect vertical center of the person’s silhouette and keep it perfectly in line with the test point.
- A person’s movement—The measurement takes some time, and the person may move during the process, which could lead to different angle estimations.

All of the above may account for a noticeable pattern in the angle estimation errors, which may mainly be caused by the person’s orientation in relation to the sensor.

An additional problem, already mentioned in Section 2.2.1, that can affect all thermal-sensor-based localization systems was observed during the post-processing of the results. Namely, the MLX90640 does not provide the temperature values for each pixel; instead, it sends raw data, which must be processed using the set of equations provided by the sensor’s manufacturer. At one point, the ambient temperature is estimated and used for pixel temperature compensation. The sensor was connected to the Raspberry Pi 4 (RPI4) minicomputer during the tests using a custom-made PCB. As the RPI4 heated up during its operation, the sensor also heated. This led to incorrect reporting of the ambient temperature (roughly 36 °C instead of 23 °C), which, in turn, led to the incorrect compensation for the pixel temperature (the human body was reported to be approximately 32 °C), which made the detection of the person in the thermal image harder and more prone to errors. Nevertheless, despite these problems, the results are satisfactory.

### 4.3. Evaluation of the VL53L5CX

**Figure 10:**
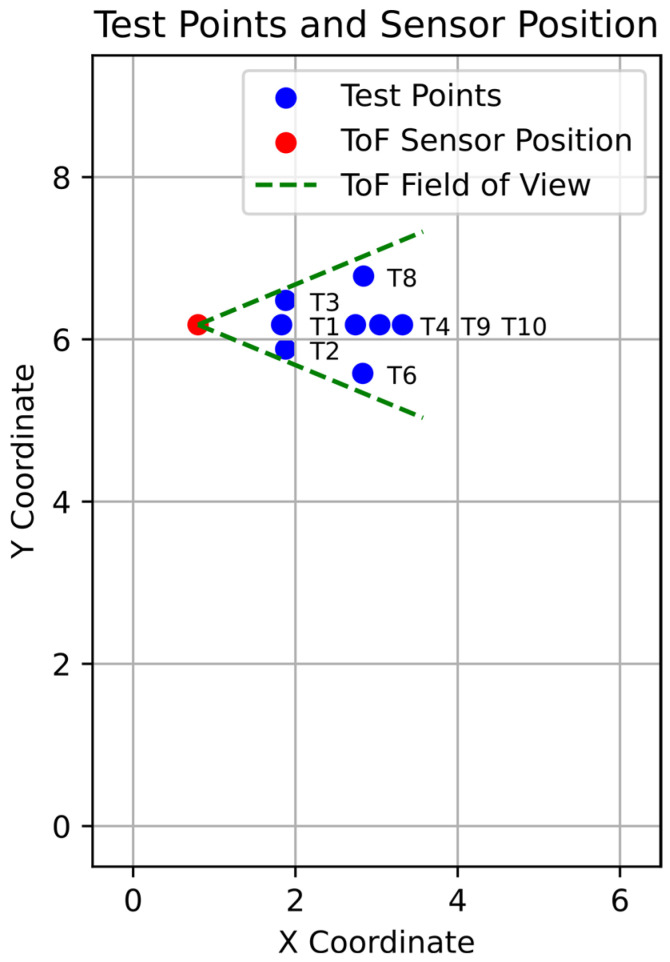
Test setup for static measurements of the VL53L5CX ToF proximity sensor.

Similarly, as for the MLX90640, the proposed angle estimation method for the VL53L5CX was tested under static conditions. Yet again, the person stood motionless in front of the sensor at eight test points, as shown in Figure 10.

At each test point, the person stood motionless until 200 results were obtained; the angle and distance values were recorded and evaluated. In Table 2, the angle calculation results are given, and in Table 3, distance measurement results are presented.

**Figure 11:**
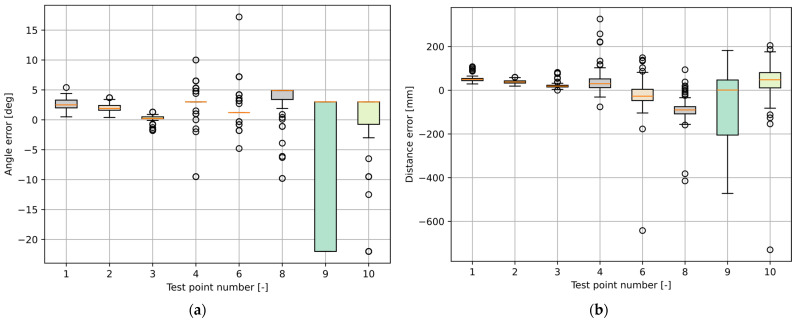
Boxplots of the VL53L5CX static measurement results for the (**a**) angle and (**b**) distance.

Boxplots of the angle estimation errors are shown in Figure 11a, and the distance measurement errors are shown in Figure 11b.

An analysis of the results shows that the error for the angle estimation and distance measurements rises with the distance from the sensor, which is consistent with the information provided in the sensor’s datasheet. The distance measurement accuracy is well within the margin defined for such conditions by the sensor’s manufacturer (11%).

The other possible sources of errors are similar to those in the case of the MLX90640 and include the sensor’s misalignment, incorrect placement of the person in front of the sensor that does not match the measured distance, and accidental movement of the person during the test.

### 4.4. Localization System Tests

#### 4.4.1. The Test Arrangement

Both hybrid solutions proposed in this paper were tested in laboratory conditions, and their performance was compared using a system based solely on BLE technology.

The test setup was arranged in accordance with Figure 7 (the location of the ToF proximity sensor was slightly changed). The Wi-Fi network was used to collect measurement results from the anchor nodes and both sensors. All of the devices were synchronized with messages transmitted over Wi-Fi. The rate of all measurements was the same, equal to 4 measurements per second.

**Figure 12:**
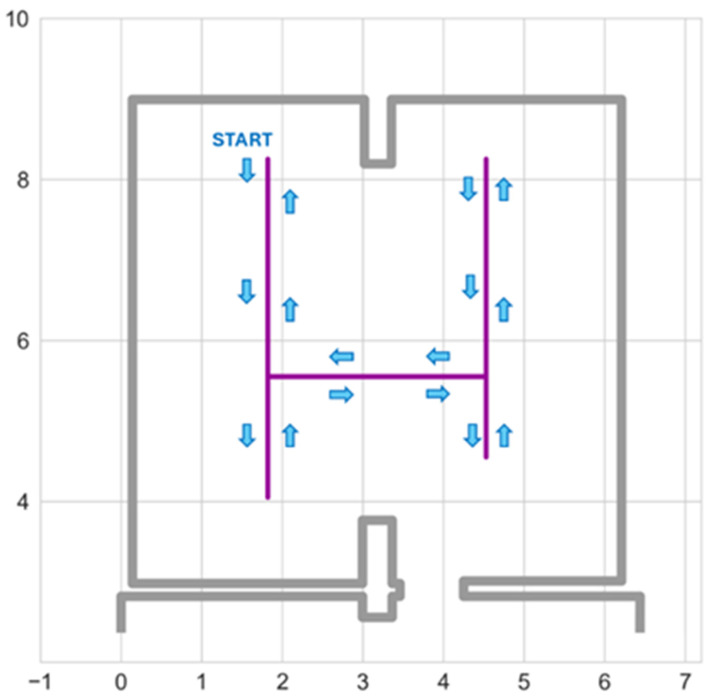
Test path, arrows indicate the direction of movement.

The tests consisted of the localization of a tag worn on a lanyard by a person who followed a predefined path (Figure 12). They maintained a constant speed while walking. The test path was composed of straight lines. The times to reach characteristic trajectory points were collected during the trials. As all of the results (the BLE signals and sensor results) were acquired at the same rate, this allowed us to determine the approximate location of the test points at which the measurements were taken. The total path length, equal to 22 m, was traveled in 63 s, so the distance between test points was close to 9 cm.

The positioning error, defined as the distance between the calculated localization and the corresponding test point, was chosen as a performance measure. Empiric cumulative distribution functions (ECDFs) were calculated for different versions of the system for the performance comparison.

#### 4.4.2. The BLE–Proximity Sensor System Test

**Figure 13:**
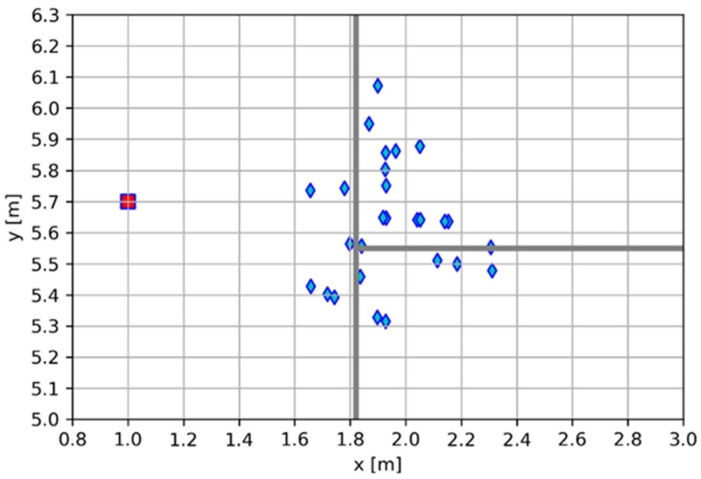
Positioning using the proximity sensor.

The proximity sensor was located close to the junction of the test path’s segments. The positioning results collected during the test are presented in Figure 13. The sensor measured the distance to the person’s body, which explains the spread of the results.

**Figure 14:**
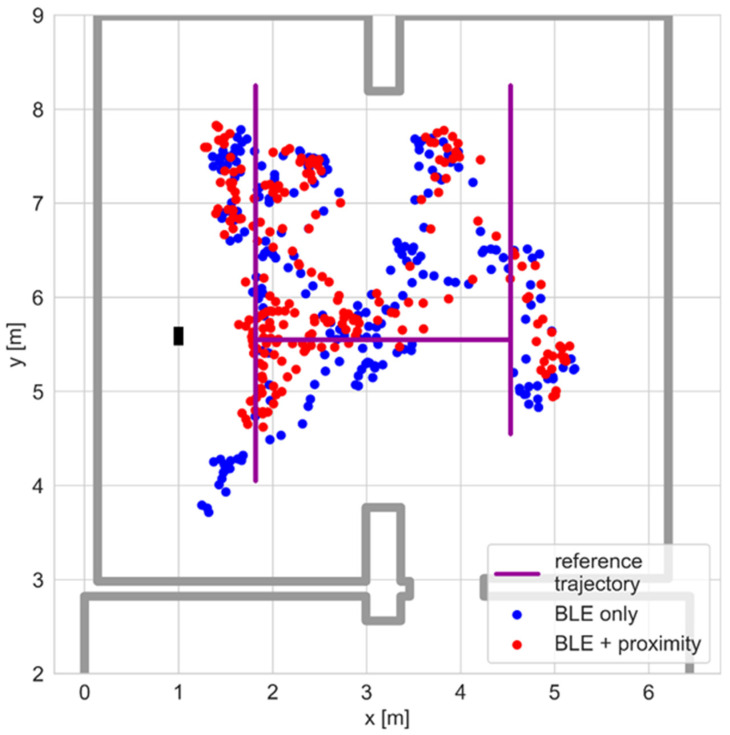
Positioning results for the BLE-proximity sensor system.

The positioning results for both system versions are presented in Figure 14. Although both algorithms processed the same BLE data, the recorded paths differed. This reflects the particle filter algorithm operation, which randomly draws new particle sets in the resampling phase.

**Figure 15:**
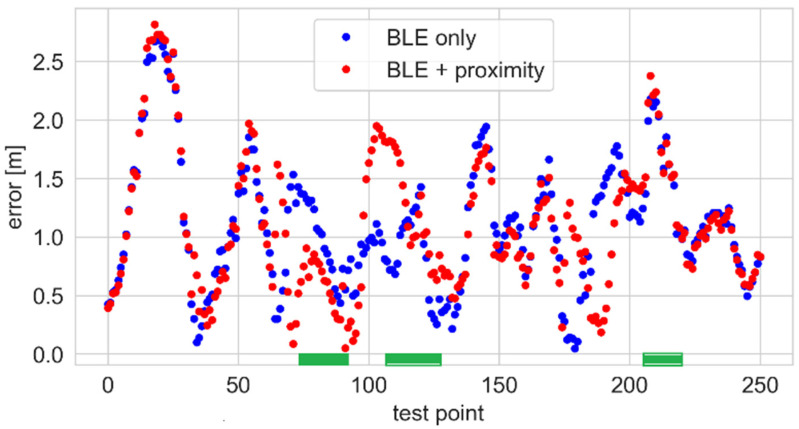
Positioning errors for the BLE-proximity sensor system.

The positioning errors are shown in Figure 15. Due to the limited sensor range, its influence can be seen only for points close to the sensor (marked green). At the start and end of the test, the errors are almost the same because both algorithms only use the BLE results.

There are several reasons why the positioning errors are relatively high. During the person’s movement, the propagation conditions between the tag and particular anchors change between LOS and non-line of sight (NLOS) as the person’s body obscures the direct path of the signal. Moreover, Bluetooth devices advertise using three channels. The anchors change the channels during the system’s operation, which additionally causes level variations. Also, the particle filter’s inertia impacts the errors, especially near the points where the person changes their direction of movement.

**Figure 16:**
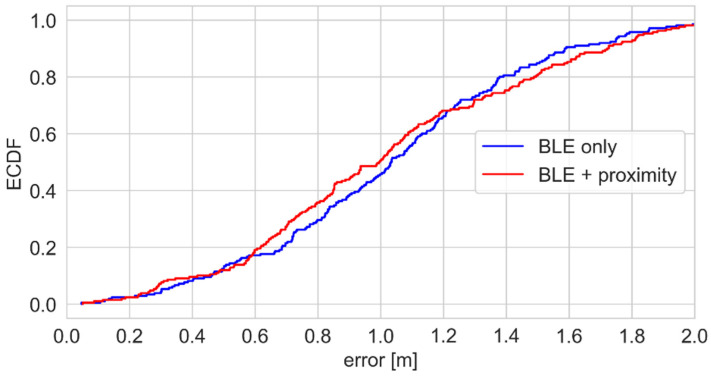
Positioning error—ECDFs for the BLE-proximity sensor system.

The empirical CDFs of the positioning errors are presented in Figure 16. The curves do not show significant differences because only part of the path is covered by the proximity sensor.

#### 4.4.3. The Hybrid BLE–Thermal Sensor Array System Test

**Figure 17:**
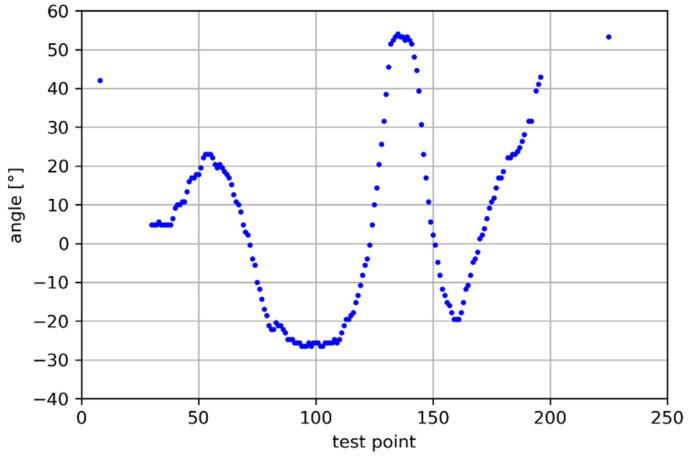
Calculated angles.

The thermal sensor array (TSA) was located close to the corner of the room to cover almost the whole test path. Figure 17 presents the angles recorded during the test. The lack of results at the beginning and the end of the test was caused by the person moving outside of the area covered by the sensor.

**Figure 18:**
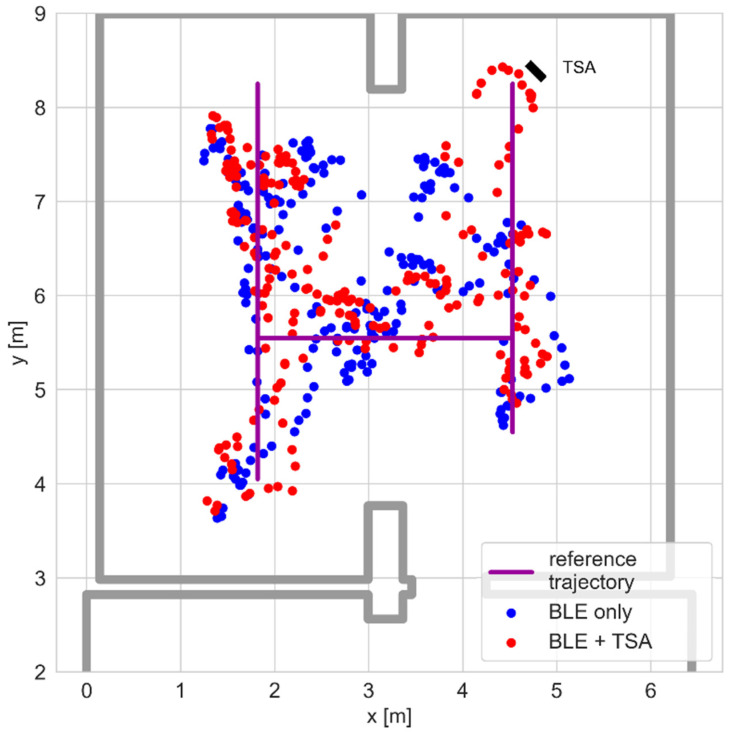
Positioning results for the hybrid BLE–thermal sensor array system.

**Figure 19:**
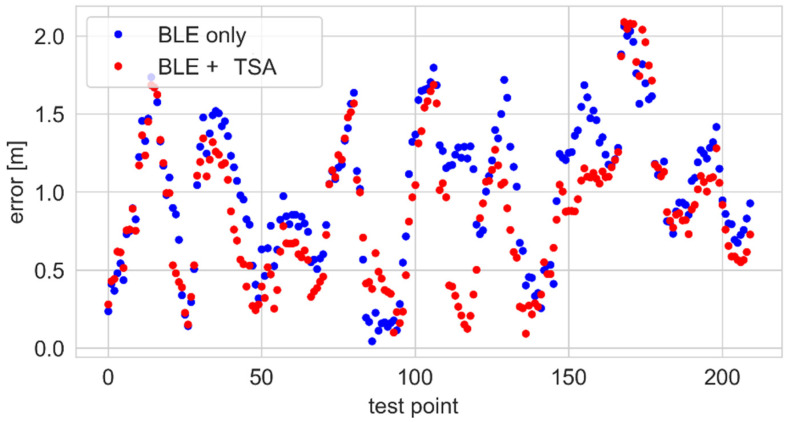
Positioning errors for the hybrid BLE–thermal sensor array system.

The trajectories recorded using the BLE and hybrid solutions are shown in Figure 18. The hybrid system’s results are better aligned with the test path. Also, the errors shown in Figure 19 are lower.

**Figure 20:**
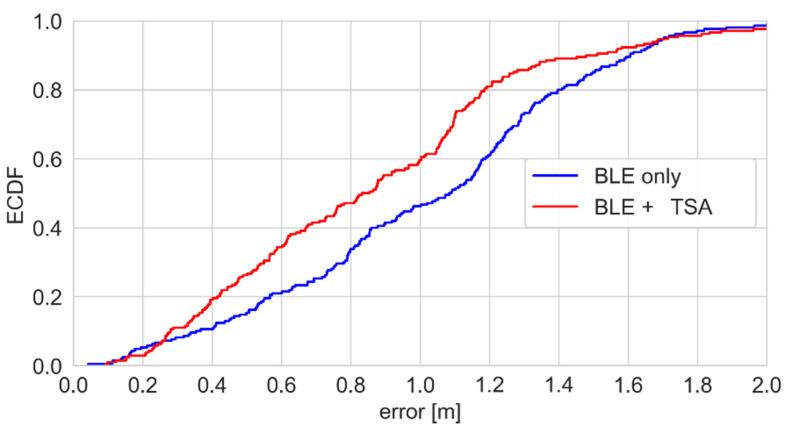
Positioning error—ECDFs for the hybrid BLE-thermal sensor array system.

According to the ECDF plots presented in Figure 20, the use of this sensor resulted in a reduction in the positioning error by several centimeters.

## 5. Conclusions

This paper presents the concept of a BLE-based system’s accuracy enhancement by using the results from proximity sensor and thermal sensor arrays. Exemplary devices available on the market were selected and used for tests in laboratory conditions. For the laser proximity sensor, the results obtained (the returning angle and the distance to the object) do not require further processing. To obtain the results from the thermal sensor array, more complex processing of the raw results is required. An example of such an algorithm is proposed in this paper. The angle determination algorithm should take into account all potential heat sources related to the person or present in the room (e.g., working computers, lamps, etc.).

The investigated sensors were integrated into the positioning system. As the positioning algorithm was based on a particle filter, the sensor results were used to update the particle weights. The performed tests confirmed the improvement in accuracy in comparison to that with the system where only the BLE results were used. However, in the case of the proximity sensor, its impact was limited due to its short operating range. Therefore, the gain in accuracy was lower in this case. 

The tested sensors are dedicated to different usage scenarios. The proximity sensor is well suited to narrow passages, whereas the thermal sensor array can cover larger areas (e.g., a whole room). Although the presented solution uses a particle filter, we believe that both sensors can be used successfully with other Bayesian positioning algorithms (e.g., based on a Kalman filter). Extending the positioning system using additional sensors increases the system’s complexity. Standalone sensors require the implementation of additional data transfer links and the provision of power. These issues can be solved by embedding the sensors into existing anchor nodes that are already equipped with links to the system controller. Also, the system’s deployment becomes more demanding, as the sensors’ fields of view should be taken into account during node installation.

The cost of the proposed solution is obviously higher, especially when thermal sensor arrays are used. At present (2025), the price of a thermal sensor array is approximately six times higher than that of a proximity sensor (EUR 30 vs. EUR 5). As the technological progress in this field is very fast, significant price reductions are expected in the future.

## Figures and Tables

**Table 1:** Results of the static test of the MLX90640 thermal sensor array (*Tn*—test point number; *d*—calculated distance between the test point and the MLX90640 sensor; $\alpha_{r}$—real angle value; $\bar{\alpha_{m}}$—mean of the obtained angle; $\bar{\alpha_{e}}$—mean error of the obtained angle; $\sigma_{\alpha }$—standard deviation of the obtained angle).

*Tn*	*d*	$\alpha_{r}$	$\bar{\alpha_{m}}$	$\bar{\alpha_{e}}$	$\sigma_{\alpha }$
[-]	[m]	[°]	[°]	[°]	[°]
0	5.5	−5.2	−2.6	−2.6	0.6
1	5.0	−13.1	−13.1	0.0	0.0
2	4.6	−22.6	−25.5	2.9	0.4
3	4.3	−33.7	−35.4	1.7	0.4
4	3.4	−30.6	−34.7	4.1	0.4
5	3.8	−17.2	−18.5	1.3	0.4
6	4.3	−6.5	−6.2	−0.3	0.0
7	4.9	1.7	4.8	−3.1	0.5
8	4.3	10.5	16.0	−5.5	0.4
9	3.9	21.6	28.6	−7.0	0.5
10	3.6	34.8	43.5	−8.7	0.8
11	3.6	2.6	7.7	−5.1	0.0
12	3.0	−9.0	−8.0	−1.0	0.5
13	2.6	−25.3	−28.1	2.8	0.5
14	1.7	−15.0	−17.2	2.2	0.5
15	2.3	4.3	7.6	−3.3	0.3
16	3.1	15.2	20.9	−5.7	0.3
17	1.1	10.3	15.4	−5.1	0.7

**Table 2:** Results of the static angle calculations of the VL53L5CX ToF proximity sensor (*Tn*—test point number; $\alpha_{r}$—real angle value; $\bar{\alpha_{m}}$—mean of the obtained angle; $\bar{\alpha_{e}}$—mean error of the obtained angle; $\sigma_{\alpha }$—standard deviation of the obtained angle).

*Tn*	$\alpha_{r}$	$\bar{\alpha_{m}}$	$\bar{\alpha_{e}}$	$\sigma_{\alpha }$
[-]	[°]	[°]	[°]	[°]
1	0.0	2.6	−2.6	0.8
2	−16.1	−14.1	−2.0	0.7
3	16.1	16.4	−0.3	0.5
4	0.0	3.0	−3.0	2.1
6	−17.2	−15.6	−1.6	1.9
8	17.1	21.0	−3.9	2.1
9	0.0	−8.8	8.8	12.5
10	0.01	−1.2	1.2	8.1

**Table 3:** Results of the static distance measurements of the VL53L5CX ToF sensor (*Tn*—test point number; $d_{r}$—real distance value; $\bar{d_{m}}$—mean of the measured distance; $\bar{d_{e}}$—mean error of the measured distance; $\sigma_{d}$—standard deviation of the measured distance).

*Tn*	$d_{r}$	$\bar{d_{m}}$	$\bar{d_{e}}$	$\sigma_{d}$
[-]	[mm]	[mm]	[mm]	[mm]
1	1030	1080	−50	11
2	1080	1118	−38	8
3	1080	1101	−21	10
4	1940	1977	−37	45
6	2030	2006	24	64
8	2040	1950	90	45
9	2520	2426	94	189
10	2240	2274	−34	115

## Data Availability

The data presented in this study are available on request from the corresponding author due to privacy reasons.
